# Changes in Cell Vitality, Phenotype, and Function of Dromedary Camel Leukocytes After Whole Blood Exposure to Heat Stress *in vitro*

**DOI:** 10.3389/fvets.2021.647609

**Published:** 2021-04-09

**Authors:** Jamal Hussen

**Affiliations:** Department of Microbiology, College of Veterinary Medicine, King Faisal University, Al-Ahsa, Saudi Arabia

**Keywords:** camel *(Camelus dromedarius)*, leukocytes, heat stress, phagocytosis, ROS, immunity

## Abstract

The dromedary camel (*Camelus dromedarius*) is well-adapted to the desert environment with the ability to tolerate increased internal body temperatures rising daily to 41–42°C during extreme hot. This study was undertaken to assess whether *in vitro* incubation of camel blood at 41°C, simulating conditions of heat stress, differently alters cell vitality, phenotype, and function of leukocytes, compared to incubation at 37°C (normothermia). Using flow cytometry, the cell vitality (necrosis and apoptosis), the expression of several cell markers and adhesion molecules, and the antimicrobial functions of camel leukocytes were analyzed *in vitro*. The fraction of apoptotic cells within the granulocytes, lymphocytes, and monocytes increased significantly after incubation of camel whole blood at 41°C for 4 h. The higher increase in apoptotic granulocytes and monocytes compared to lymphocytes suggests higher resistance of camel lymphocytes to heat stress. Functionally, incubation of camel blood at 41°C for 4 h enhanced the phagocytosis and ROS production activities of camel neutrophils and monocytes toward *S. aureus*. Monocytes from camel blood incubated at 41°C for 4 h significantly decreased their expression level of MHC class II molecules with no change in the abundance of CD163, resulting in a CD163^high^ MHC-II^low^ M2-like macrophage phenotype. In addition, heat stress treatment showed an inhibitory effect on the LPS-induced changes in camel monocytes phenotype. Furthermore, *in vitro* incubation of camel blood at 41°C reduced the expression of the cell adhesion molecules CD18 and CD11a on neutrophils and monocytes. Collectively, the present study identified some heat-stress-induced phenotypic and functional alterations in camel blood leukocytes, providing a paradigm for comparative immunology in the large animals. The clinical relevance of the observed changes in camel leukocytes for the adaptation of the camel immune response to heat stress conditions needs further *in vitro* and *in vivo* studies.

## Introduction

Dromedary camels (*Camelus dromedarius*) are economically important arid-desert-adapted livestock with a relatively high capability of tolerating extreme hot conditions (1–3). They are characterized by their heterothermy, enabling them to tolerate body temperatures rising daily to 41–42°C, compared with normal 37–38°C (4–6). Detailed genomic studies revealed the existence of complex adaptations of camels to the harsh desert environment, including specific heat stress responses (7). The adaptation of several cellular systems of camels to heat stress conditions has been investigated using *in vivo* and *in vitro* studies. Early studies on the impact of heat stress on camel lymphocytes revealed higher protein synthesis competence for camel lymphocytes compared to human lymphocytes (8). Similarly, camel platelets showed relatively higher resistance to temperatures of 43–45°C compared to human platelets (9). Even extreme temperatures of 50°C were not sufficient to disrupt the function of camel platelets while causing damages to human platelets (9). An *in vitro* study investigated the effect of heat stress on the survival of the camel cell line Dubca cells and identified this cell line as heat stress resistant being able to survive the 42°C heat treatment in comparison to a mouse fibroblast cell line (1). In addition, the cellular thermotolerance in camels seems to be cell-type specific, as camel oocytes and cumulus cells showed different capabilities to tolerate acute (45°C for 2 h) and chronic (45°C for 20 h) heat shock *in vitro* (10, 11). Cellular thermo-tolerance in camels is regulated by the expression of a protein family called heat shock proteins (HSPs), which mediate maintenance of cell viability and inhibition of apoptosis-mediated cellular death (11). Upon exposure to thermal stress, camel lymphocytes showed a significant upregulation of the heat-shock protein 73 (8).

Recent studies in camel immunology have enabled the characterization of several subpopulations of camel leukocytes, including camel monocyte subsets, some subsets of camel lymphocytes, and camel neutrophilic granulocytes (12–18). Neutrophils and monocytes are circulating myeloid immune cells with key roles in innate immunity to bacterial infections. The anti-microbial activity of neutrophils and monocytes is mainly mediated by phagocytosing bacteria and the subsequent killing of ingested bacteria by means of the produced oxygen metabolites (19). In addition to their ability to ingest and kill bacteria, monocytes are the main source of tissue macrophages upon leaving the bloodstream and migration to tissue (19–21). The expression of the cell markers CD163 and major histocompatibility (MHC) class II molecules are considered as markers for the functional subtype of monocytes during their differentiation into macrophages (22–25). CD163 is a scavenger receptor for haptoglobin–hemoglobin complexes that is mainly expressed on monocytes and macrophages and is considered as a marker for anti-inflammatory functional subtype (M2) of these cells (26, 27). In contrast, the antigen-presenting receptors MHC class II molecules are markers for inflammatory monocytes and macrophages (M1) (16, 28). The cell adhesion molecule lymphocyte function antigen-1 (LFA-1), which is expressed as a dimer of CD11a and CD18 on all leukocytes play an essential role in leukocyte adhesion and migration as well as the adhesion of phagocytes to bacterial surfaces (29, 30).

As no published studies have yet examined the physiological impact of heat stress on the phenotype and function of cellular immunology in dromedary camels, the current study investigated the time-dependent heat stress-induced changes in cell vitality, phenotype, and function of leukocytes separated from camel blood exposed to heat stress (41°C) compared to normal temperatures (37°C).

## Materials and Methods

### Animals and Blood Sampling

Blood samples were collected from five apparently healthy female dromedary camels *(Camelus dromedarius)* aged between 10 and 14 years. The animals were housed and fed in free stalls at the Camel Research Center, King Faisal University, Al-Ahsa, Saudi Arabia. Blood was obtained by venipuncture of the external jugular vein (vena jugularis externa) into vacutainer tubes containing EDTA (Becton Dickinson, Heidelberg, Germany). To avoid the impact of environmental heat stress on the current *in vitro* study, the experiments were carried out in October 2020 when the camels were not exposed to heat stress conditions. The average daily temperature during the sampling period ranged between a minimum temperature of 18°C and a maximum temperature of 27°C. The Ethics Committee at King Faisal University, Saudi Arabia approved all experimental procedures and management conditions used in this study (Permission number: KFU- KFU-REC/2020-09-25).

### *In vitro* Heat Stress Treatment of Camel Whole Blood and Separated Leukocytes

The *in vitro* heat treatment of camel whole blood was performed as previously described (31) with some modifications. Blood samples (1 mL) were diluted with 1 mL RPMI medium supplemented with 10% heat-inactivated fetal bovine serum, 2 mM L-glutamine, 100 U/mL penicillin, and 10 mg/mL streptomycin (Gibco Laboratories, Carlsbad, CA) in sterile 12 × 75 mm glass tubes (BD Biosciences, San Jose, California, USA), and the diluted samples were incubated either at 37°C (normal temperature) or 41°C (heat stress) for different times (1, 2, or 4 h). For heat treatment of separated cells, leukocyte cell suspension (5 x 10^6^ cells/mL) in RPMI medium supplemented with 10% heat-inactivated fetal bovine serum, 2 mM L-glutamine, 100 U/mL penicillin, and 10 mg/mL streptomycin (Gibco Laboratories, Carlsbad, CA) were incubated either at 37 or 41°C for 4 h.

### *In vitro* Stimulation of Camel Whole Blood With Lipopolysaccharide

Whole blood stimulation with lipopolysaccharide (LPS) was performed as previously described (32). Briefly, blood samples (*n* = 5) were diluted (1:2) with RPMI medium supplemented with 10% heat-inactivated fetal bovine serum, 2 mM L-glutamine, 100 U/mL penicillin, and 10 mg/mL streptomycin (Gibco Laboratories, Carlsbad, CA) and were stimulated with 1 μg/mL LPS from *E.coli* (Sigma-Aldrich, Germany) for 4 h at 37 or 41°C. After incubation, blood samples were diluted (1:4) with phosphate buffer saline (PBS) and centrifuged at 10°C for 10 min at 1,000 × *g*. After removing the supernatant, the cell pellet was resuspended in PBS for further analysis.

### Leukocytes Separation

Separation of camel leukocytes was done after hypotonic lysis of erythrocytes (33). Blood samples were diluted with PBS (1:4) and centrifuged at 1,000×*g* for 10 min without break. After careful removal of blood plasma, the erythrocytes were lysed by adding 5 mL distilled water for 20 s and subsequent addition of 5 mL double concentrated PBS to restore tonicity. After centrifugation at 500×*g* for 10 min with break, the cell pellet was resuspended. The erythrolysis was repeated (usually twice) until the complete removal of red blood cells (clear white pellet of leukocytes). Subsequently, the cells were suspended in 10 mL PBS and washed two times (250×*g* and 100×*g* for 10 min each) and finally adjusted to 5 × 10^6^ cells/mL in RPMI medium.

### Leukocyte Viability Assay

Cell viability of blood leukocytes was analyzed using the dye exclusion assay (34). Separated leukocytes were incubated (in duplicates) with the DNA-binding dye propidium iodide (PI; 2 μg/mL, Calbiochem, Germany) and labeled cells were analyzed by flow cytometry (Accuri C6 flow cytometer, BD Biosciences). PI uptake vs. exclusion was used to discriminate dead necrotic cells with permeable plasma membranes (PI-positive) from live cells with intact membranes (PI-negative). For the analysis of cell apoptosis, the mitochondrial membrane potential (MMP) probe JC-1 (5,5′,6,6′-tetrachloro-1,1′,3,3′-tetraethylbenzimidazolcarbocyanine iodide) was used as previously described (35, 36). Separated camel leukocytes (100 μL) were plated in a 96-well microtiter plate at a density of 5 × 10^6^ cell/mL in RPMI cell culture medium supplemented with 10% heat-inactivated fetal bovine serum, 2 mM L-glutamine, 100 U/mL penicillin, and 10 mg/mL streptomycin (Gibco Laboratories, Carlsbad, CA). In each well, 100 μL JC-1 solution (2 μmol/L final concentration) was added and the plates were incubated for 15 min in a humidified atmosphere (5% CO2) at 37°C. After two washing steps with PBS, cells were suspended in 200 μL PBS, transferred to flow cytometer tubes, and acquired with the BD Accuri C6 flow cytometer. In apoptotic cells (with low MMP), JC-1 forms monomers, which emit in the green fluorescence channel (FL-1) at 525 nm upon excitation at the 488 nm. In normal cells (with high MMP), JC-1 forms aggregates, which display an orange fluorescence (585 nm, detected in FL-2).

### Phagocytosis Assay

Heat killed *staphylococcus aureus* (*S. aureus*) bacteria (Pansorbin, Calbiochem, Merck, Nottingham, UK) were labeled with fluoresceinisothiocyanate (FITC, Sigma-Aldrich, St. Louis, Missouri, USA) according to manufacturer instructions. FITC-conjugated and heat-killed *S. aureus* bacteria were washed thoroughly, suspended in RPMI medium (2 × 10^8^ bacteria/mL), and stored in single-use aliquots at −80 °C. Leukocytes separated from heat-stressed whole blood or heat-stressed leukocytes were plated in 96 well-plates (1 × 10^6^/well) in duplicates and incubated with FITC-conjugated *S. aureus* (30 bacteria/cell) for 30 min (37°C, 5% CO2). Control samples were incubated without bacteria. After incubation, samples were washed twice with PBS and analyzed by flow cytometry. Phagocytic activity of monocytes and neutrophils was defined as the percentage of green fluorescing cells among viable cells. Mean green fluorescence intensity (MFI) of phagocytosis-positive cells was measured as an indicator for the number of bacteria phagocytosed by each cell.

### Generation of Reactive Oxygen Species (ROS)

ROS generation was measured in 96-well round-bottom microtiter plates (Corning, NY, USA) as previously described (37). Leukocytes separated from heat-stressed whole blood or heat-stressed leukocytes (1 × 10^6^/well in RPMI medium) were incubated in duplicates for 20 min (37°C, 5% CO_2_) with heat-killed *S. aureus* (30 bacteria/cell) in the presence of the ROS-sensitive dye dihydrorhodamine (DHR)-123 (1 μg/mL final, Mobitec, Goettingen, Germany). After incubation, labeled cells were washed with PBS and the relative amount of generated ROS was determined by flow cytometry (Accuri C6 flow cytometer, BD Biosciences) after the acquisition of 100 000 events (gated leukocytes).

### Membrane Immunofluorescence and Flow Cytometry

The expression of the monocytes markers CD163 and major histocompatibility complex (MHC) class II molecules as well as the cell adhesion molecules CD11a and CD18 was analyzed using membrane immunofluorescence test as previously described (38). Separated leukocytes (4 × 10^5^) were incubated (15 min; 4°C) with unlabeled primary monoclonal antibodies (mAbs) specific for the cell markers CD14, CD163, and MHC-class II or with directly labeled mAbs to the cell adhesion molecules CD11a and CD18 (16). After two washing steps, the cells labeled with primary anti-CD14, anti-CD163, or anti-MHC class II molecules (mAbs) were incubated with mouse secondary antibodies (IgG1, IgG2a; Invitrogen) labeled with different fluorochromes. Mouse isotype control antibodies (Becton Dickinson Biosciences) were also included. Washed cells were analyzed using the Accuri C6 flow cytometer (BD Biosciences). At least 100 000 total leukocytes were collected and analyzed with the CFlow Software, Version 1.0.264.21 (BD Biosciences).

### Statistical Analyses

Statistical analysis was carried out using the software Prism (GraphPad software version 5). Results were presented as means ± S.E. of the mean (SEM). Differences between the means were tested using the one-factorial analysis of variance (ANOVA) in combination with the Bonferroni's method for a pairwise comparison of the means. Results were considered statistically significant if the *p*-value was <0.05.

## Results

### Impact of Heat Stress on Cell Viability of Camel Blood Leukocyte Subpopulations

**Figure 1:**
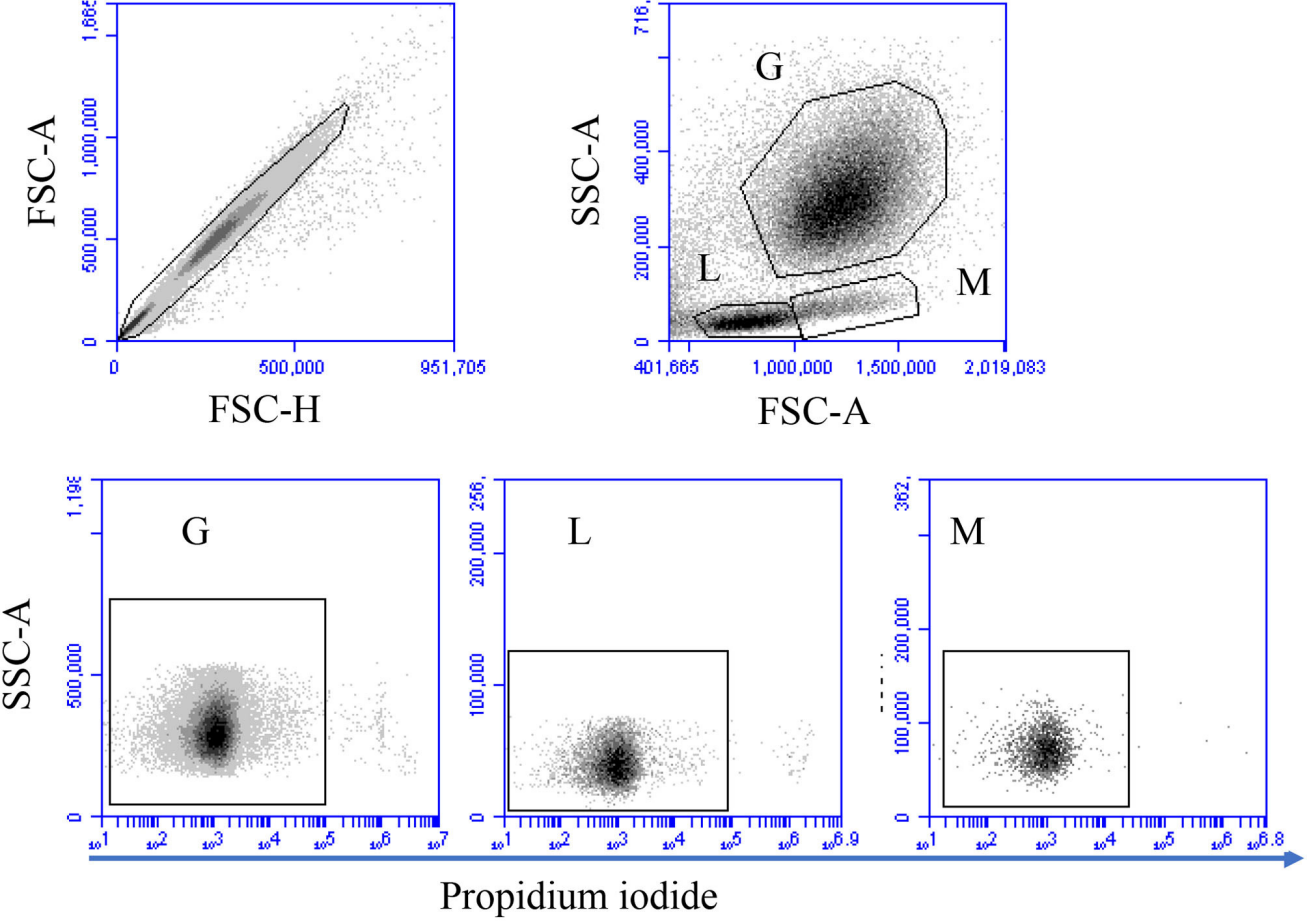
Gating strategy for flow cytometric analysis of cell necrosis. Separated blood leukocytes were loaded with the DNA-sensitive dye propidium iodide (PI) and labeled cells were analyzed by flow cytometry. Cell duplicates were excluded using a FSC-H against FSC-A dot plot. After gating on single cells, camel granulocytes (G), lymphocytes (L), and monocytes (M) were identified based on their FSC and SSC properties. Viable (PI-negative cells) and necrotic (PI-permeable cells) were identified according to their staining with PI in a SSC-A against PI (FL-3).

**Figure 2:**
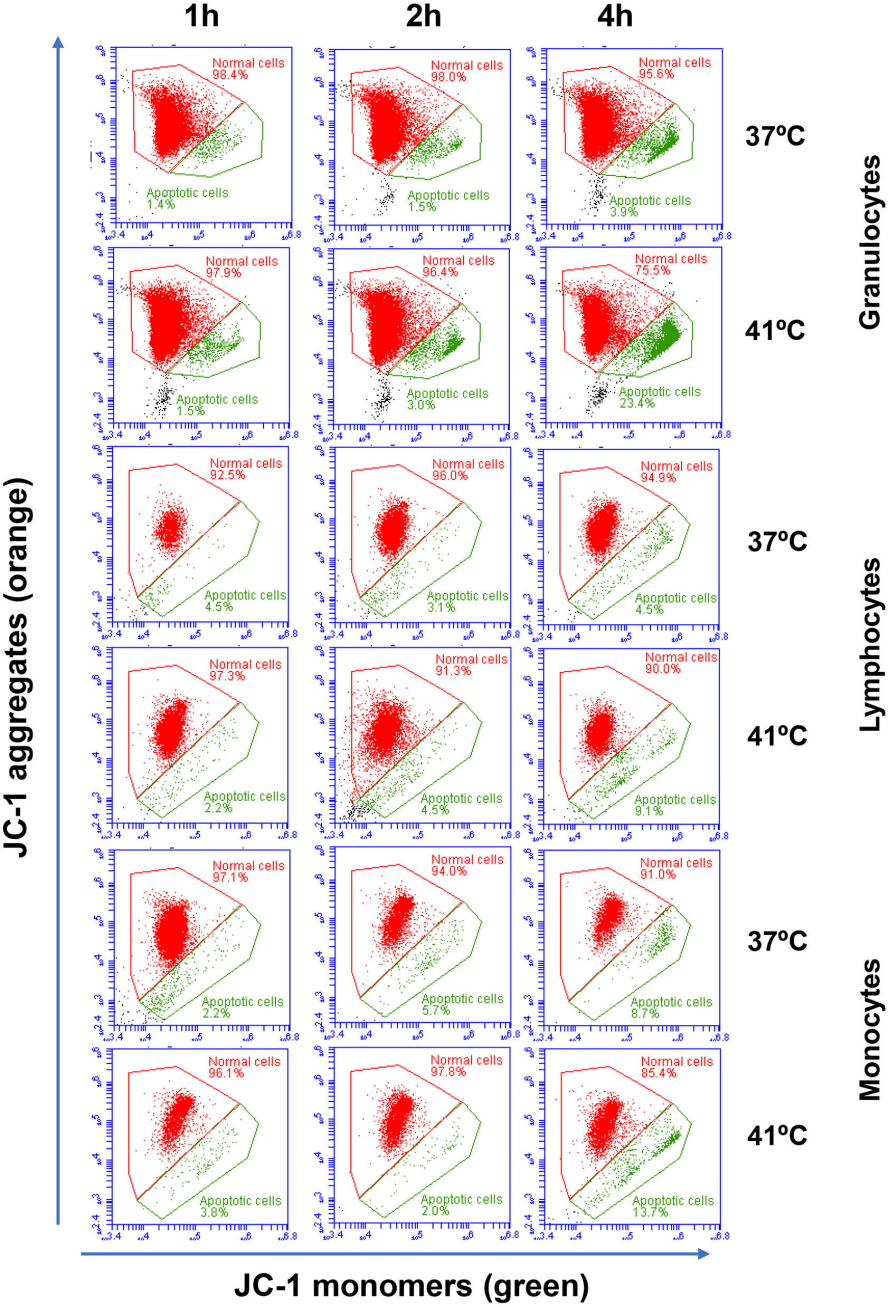
Flow cytometric analysis of cell apoptosis. Separated blood leukocytes were loaded with the mitochondrial membrane potential (MMP) probe JC-1. After setting gates on camel granulocytes, lymphocytes, and monocytes based on their FSC and SSC characteristics and the exclusion of cell duplicates, the fractions of normal viable cells (orange JC-1 aggregates detected in FL-2) and apoptotic cells (green JC-1 monomers detected in FL-1) were calculated for blood incubated at 37 and 41°C for 1, 2, and 4 h.

**Figure 3:**
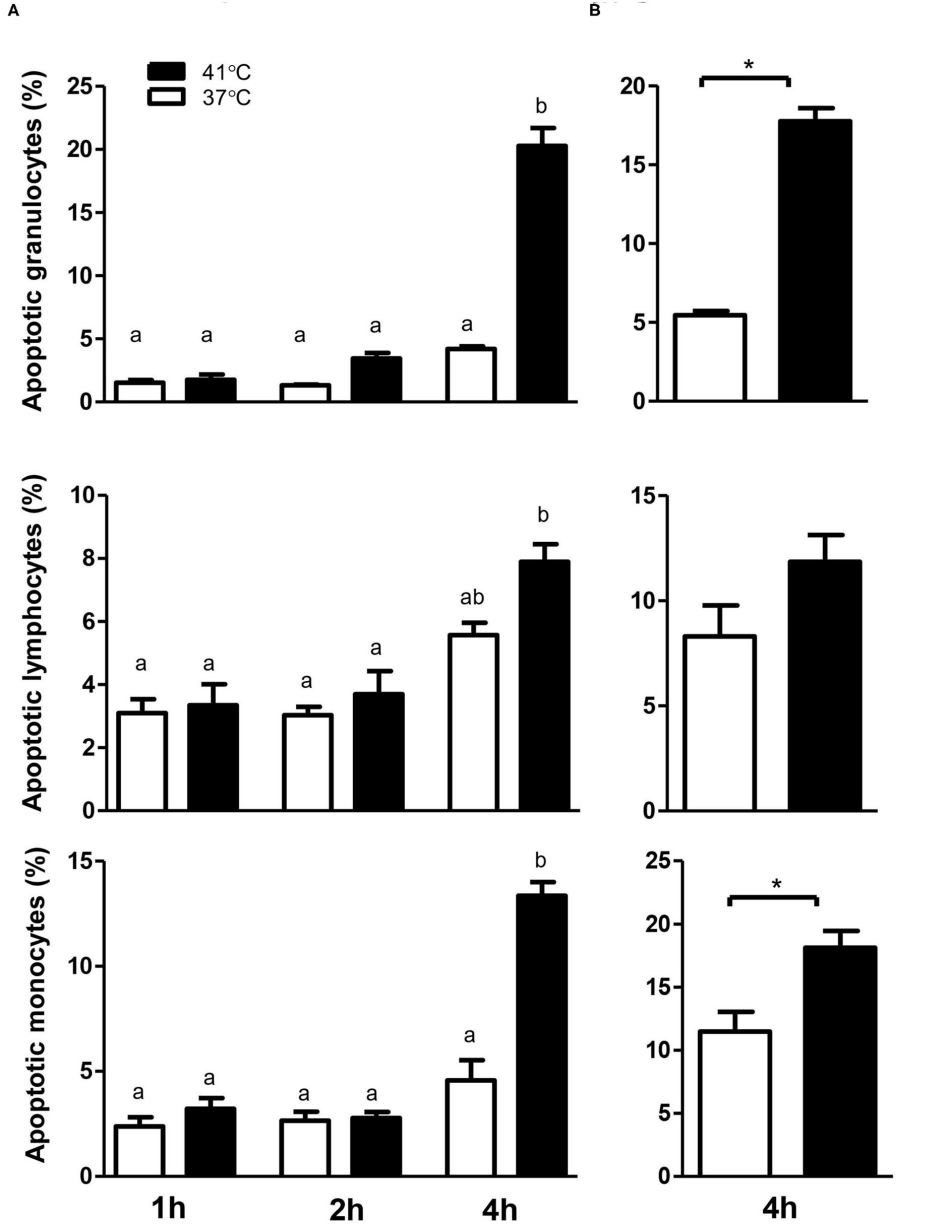
The impact of heat stress on leukocyte cell apoptosis. Whole camel blood **(A)** or separated camel leukocytes **(B)** were incubated at 37 or 41°C for different times. The cells were loaded with the mitochondrial membrane potential (MMP) probe JC-1 and were analyzed by flow cytometry. The percentages of apoptotic cells were calculated for gated camel granulocytes, lymphocytes, and monocytes and presented as mean ± SEM. Different lower case letters indicate a significant difference between the groups as analyzed using one-way ANOVA (*p* < 0.05). For separated leukocytes, the student *t*-test was used for comparison between the means of cells incubated at 37 and 41°C (* indicates *p* < 0.05).

The analysis of cell necrosis using the DNA sensitive dye propidium iodide (Figure 1) revealed no impact of heat treatment of whole blood on the necrotic cell death of camel leukocytes. The number of total leukocytes in blood and the fractions of granulocytes, lymphocytes, and monocytes populations did not change significantly (*p* > 0.05) after incubation of blood at 37 or 41°C for 1, 2, or 4 h (Table 1). The identification of apoptotic cells using the MMP probe JC-1, however, revealed significant differences between the two heat treatments of camel blood (37 and 41°C) (Figure 2). Incubation of camel blood for 1 or 2 h at 41°C did not result in a significant change in the fraction of apoptotic cells within the granulocytes, lymphocytes, or monocytes populations (Figure 3A). Incubation of blood for 4 h at 41°C, however, resulted in a significant (*p* < 0.05) increase in the fraction of apoptotic granulocytes (20.3 ± 1.4 % compared to 3.5 ± 0.4 % after 2 h incubation at 41°C), lymphocytes (7.9 ± 0.6 % compared to 3.7 ± 0.6 % after 2 h incubation at 41°C), and monocytes (13.4 ± 2.0 % compared to 2.8 ± 0.3 % after 2 h incubation at 41°C). In addition, the fractions of apoptotic cells within the granulocytes and monocytes populations, but not the lymphocytes population, were significantly higher in blood incubated for 4 h at 41°C compared to blood incubated at 37°C for the same time (*p* < 0.05). For camel granulocytes, monocytes, and lymphocytes, the fraction of apoptotic cells did not change significantly (*p* > 0.05) after incubation of whole blood at 37°C for 1, 2, or 4 h (Figure 3A). When the heat stress treatment was performed on separated leukocytes (from the beginning) instead of whole blood, similar increase in the percentage of apoptotic granulocytes and monocytes was observed after 4 h of incubation at 41°C compared to cells incubated at 37°C (Figure 3B). The percentage of apoptotic lymphocytes, however, did not differ significantly between cells incubated at 41°C and those incubated at 37°C. Compared to the starting cell number at the beginning (5 × 10^5^ cells/well), 4 h-incubation of separated leukocytes at 37°C (4.8 ± 0.1 × 10^5^ cells/well) or 41°C (4.7 ± 0.1 x 10^5^ cells/well) did not induce a significant change in the number of viable PI-negative cells.

**Table 1:** The impact of heat treatment on leukocyte count, their differential composition, and the percentage of viable (non-necrotic) cells.

**Incubation temperature**	**37°C**			**41°C**		
**Incubation time**	**1 h**	**2 h**	**4 h**	**1 h**	**2 h**	**4 h**
WBC cell/μl (×10^3^)	13.4, 1.8	12.9, 0.8	13.1, 1.0	13.7, 1.0	13.3, 1.1	12.8, 0.9
Neutrophils % of WBC	77.1, 0.6	76.8, 0.4	76.4, 0.2	76.5, 0.8	76.3, 0.4	69.6, 0.6
Viable (PI^−^) cells (%)	96.9, 1.4	95.1, 1.3	96.4, 1.7	95.2, 1.7	95.9, 1.7	94.6, 1.9
Lymphocytes % of WBC	19.2, 0.6	19.1, 0.8	19.8, 0.5	19.5, 0.4	19.3, 0.5	24.6, 0.9
Viable (PI^−^) cells (%)	95.8, 1.1	96.7, 1.7	96.6, 1.6	95.7, 1.3	94.0, 1.4	93.8, 1.7
Monocytes % of WBC	2.6, 0.3	2.5, 0.3	2.4, 0.2	2.4, 0.1	2.6, 0.2	3.1, 0.3
Viable (PI^−^) cells (%)	96.7, 1.1	95.8, 1.1	95.5, 1.3	95.5, 1.2	96.4, 1.3	94.0, 1.4

*Microscopic estimation of the total leukocyte count (WBC) was performed using Neubauer counting chamber after incubation of blood with Türk solution for erythrolysis. PI, propidium iodide*.

### Impact of Heat Stress on Phagocytosis Activity of Camel Blood Monocytes and Neutrophils

**Figure 4:**
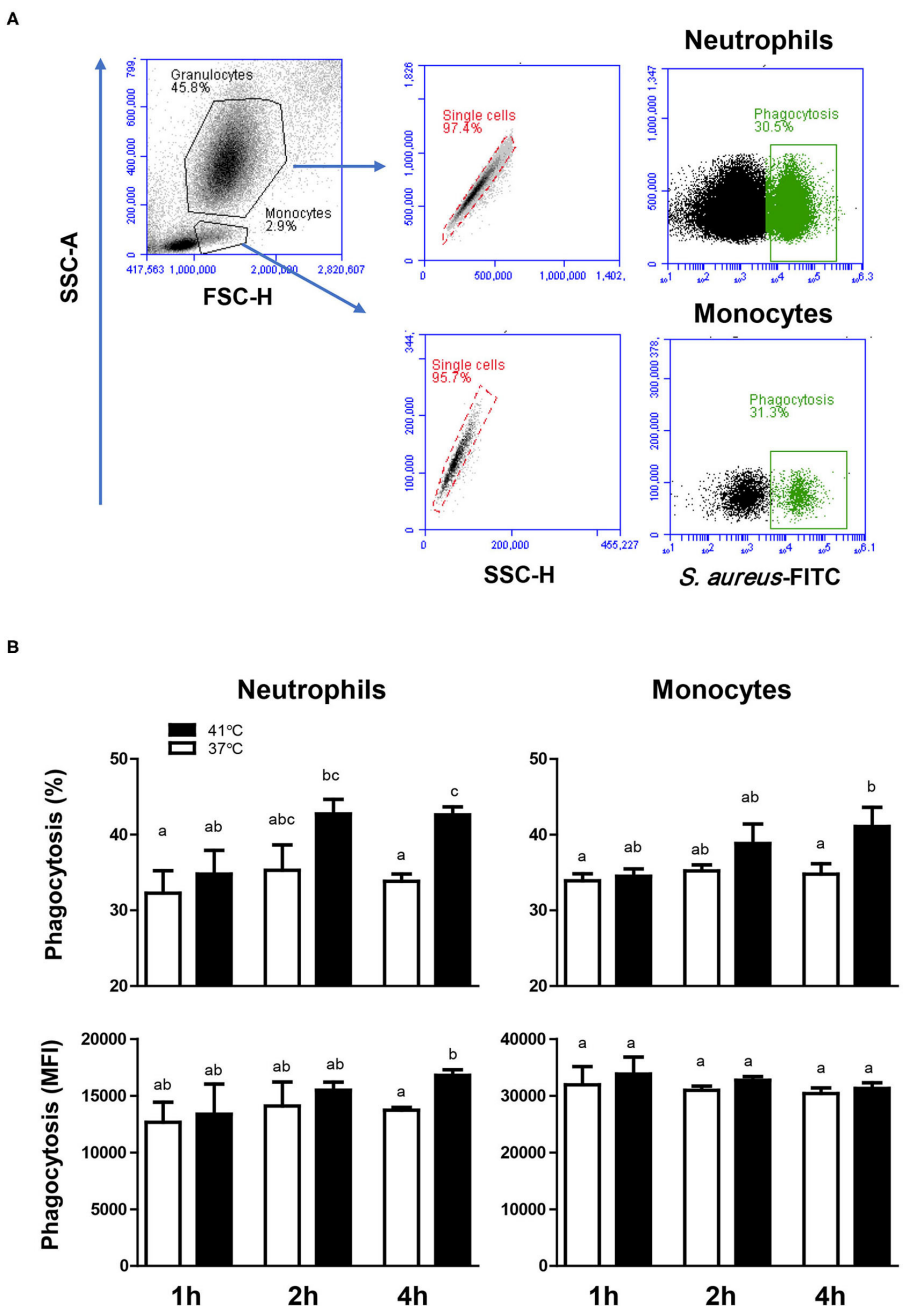
The impact of heat stress on bacterial phagocytosis by camel neutrophils and monocytes. **(A)** Separated camel leukocytes were incubated with heat-killed FITC-labeled *S. aureus* bacteria and their phagocytosis activity was analyzed by flow cytometry. After gating on neutrophils and monocytes, the percentage of FITC-positive, phagocytosis-positive cells and the phagocytosis capacity (indicating the number of bacteria phagocytosed by each cell as measured by MFI of phagocytosis-positive cells) were determined for each cell type. **(B)** Data were presented as means ± SEM for blood incubated at 37 and 41°C for 1, 2, and 4 h. Different lower case letters indicate a significant difference between the groups as analyzed using one-way ANOVA (*p* < 0.05).

For both neutrophils and monocytes and for all incubation times (1, 2, or 4 h), neither the percentage of phagocytosis-positive cells nor the phagocytosis capacity (number of bacteria phagocytosed by each cell as measured by the MFI of phagocytosis-positive cells) showed a significant change (*p* > 0.05) after incubation of whole blood at 37°C (Figure 4). The comparison between blood incubated at 37 and 41°C, however, revealed significantly (*p* < 0.05) higher fraction of phagocytosis-positive cells (for neutrophils and monocytes) and higher (*p* < 0.05) phagocytosis capacity (only for neutrophils) in blood incubated for 4 h at 41°C than in blood incubated at 37°C for the same time (Figure 4). Although heat treatment of separated leukocytes (instead of whole blood) for 4 h at 41° resulted in a significant increase (*p* < 0.05) in the phagocytosis activity of monocytes (56.8 ± 4.2% vs. 45.8 ± 2.6% for cells incubated at 37°C), no significant (*p* > 0.05) change was observed in the phagocytosis activity of neutrophils (52.1 ± 2.8% vs. 48.8 ± 2.7% cells incubated at 37°C) compared to cells incubated at 37°C.

### Impact of Heat Stress on ROS Production Activity of Camel Blood Monocytes and Neutrophils

**Figure 5:**
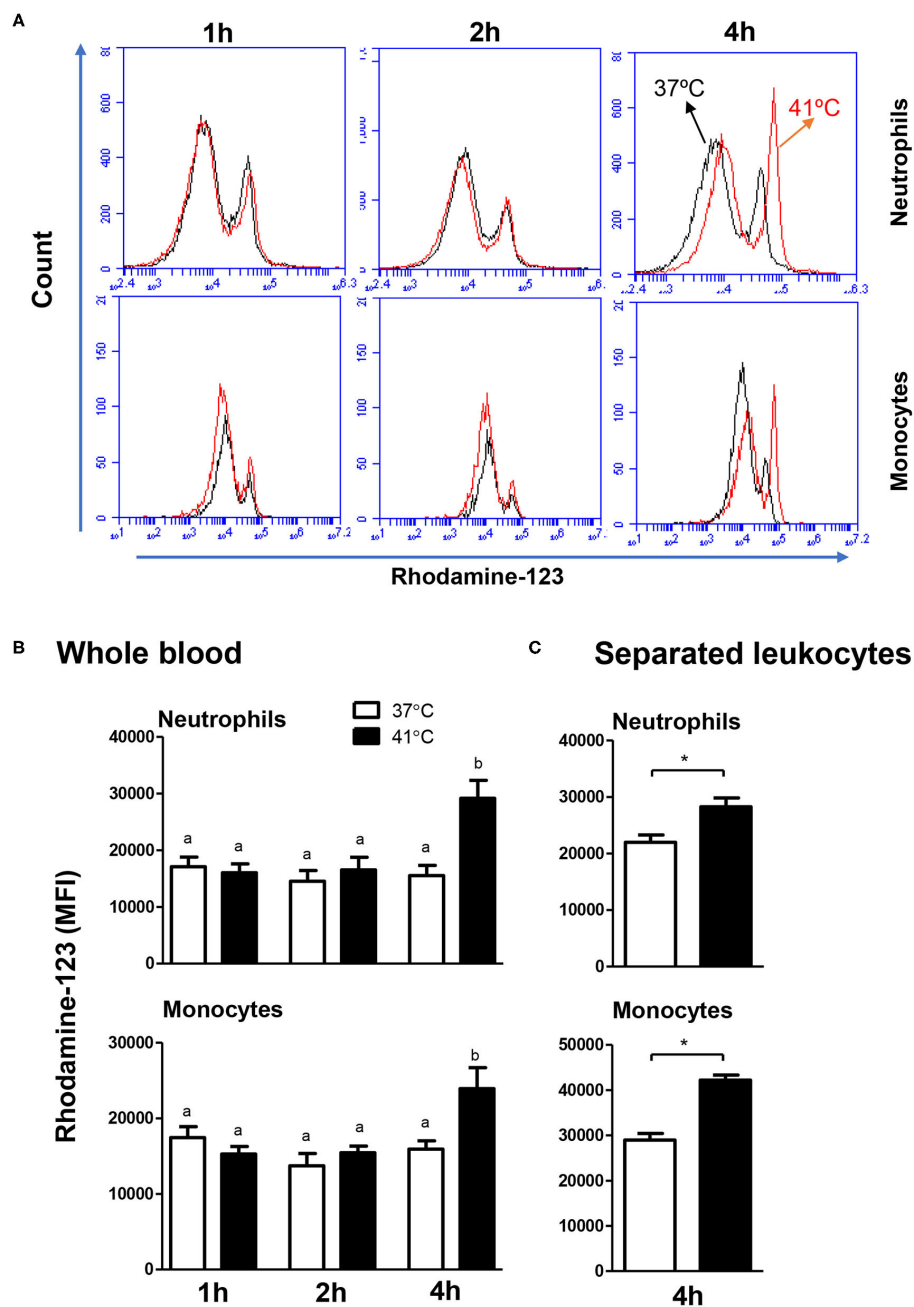
The impact of heat stress on *S. aureus*-induced ROS-response of camel neutrophils and monocytes. **(A)** Flow cytometric analysis of ROS production by camel neutrophils and monocytes. Separated camel leukocytes were stimulated with heat-killed *S. aureus* bacteria in the presence of the ROS-sensitive dye dihydrorohdamin-123 (DHR-123) and the reactive oxygen-dependent generation of rhodamine-123 was analyzed by flow cytometry. After gating on neutrophils and monocytes, the mean fluorescence intensity (MFI) of rhodamine-123 was presented in a count/FL-1 histogram. **(B)** MFI values of rhodamine-123 were presented for granulocytes and monocytes (mean ± SEM) from whole blood incubated at 37 or 41°C for 1, 2, and 4 h. Different lower case letters indicate a significant difference between the groups, as analyzed using one-way ANOVA (*p* < 0.05). **(C)** MFI values of rhodamine-123 were presented for granulocytes and monocytes (mean ± SEM) from separated leukocytes incubated at 37 or 41°C for 4 h. For separated leukocytes, the student *t*-test was used for comparison between the means of cells incubated at 37 and 41°C (* indicates *p* < 0.05).

The ROS response of both neutrophils and monocytes did not change significantly (*p* > 0.05) after 1, 2, or 4 h of incubation of whole blood at 37°C. For blood samples incubated for 4 h at 41°C, neutrophils and monocytes produced significantly (*p* < 0.05) more ROS compared to the ROS response after 1 or 2 h of incubation at the same temperature (Figures 5A,B). In addition, incubation of blood for 4 h resulted in significantly (*p* < 0.05) higher ROS response in neutrophils and monocytes, when blood was incubated at 41°C compared to 37°C (Figures 5A,B). For separated leukocytes (instead of whole blood), heat treatment for 4 h at 41°C induced a similar increase in ROS production in monocytes (42,226 ± 4,851 vs. 35,589 ± 1,492 for cells incubated at 37°C) and neutrophils (28,263 ± 3,203 vs. 21,978 ± 1,281 for cells incubated at 37°C) in comparison to cells incubated at 37°C for the same time (Figure 5C).

### Heat Stress Modulates the Phenotype of Blood Monocytes

**Figure 6:**
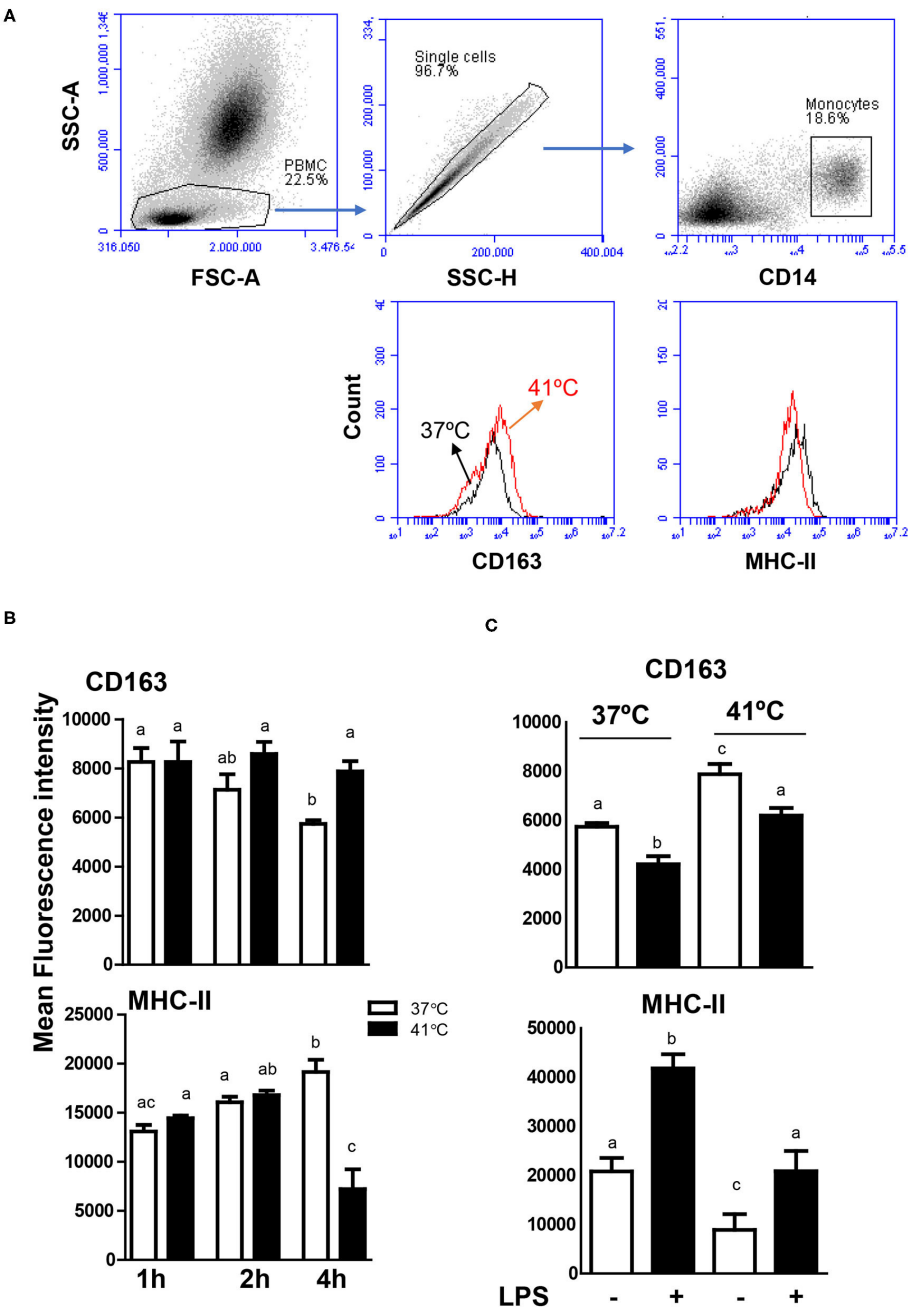
Modulatory effects of heat stress on the phenotype of camel monocytes. **(A)** Gating strategy for the flow cytometric analysis of expression of CD163 and MHC class II molecules on camel monocytes. Separated camel leukocytes were labeled with monoclonal antibodies to CD14, CD163, and MHC class II molecules and analyzed by flow cytometry. Monocytes were identified as CD14-positive cells within mononuclear cells after excluding cell duplets using SSC-A/SSC-H dot plot. The expression levels of CD163 and MHC class II molecules were presented as histograms. **(B)** The MFI values of CD163 and MHC class II molecules were calculated and presented (mean ± SEM) for blood incubated at 37 and 41°C for 1, 2, and 4 h. Different lower case letters indicate significant difference between the groups as analyzed using one-way ANOVA (*p* < 0.05). **(C)** Impact of heat stress on LPS-induced change in monocyte phenotype. Leukocytes were separated from camel blood incubated at 37 or 41°C with or without LPS stimulation. The MFI values of CD163 and MHC class II molecules were calculated and presented (mean ± SEM). Different lower case letters indicate a significant difference between the groups, as analyzed using one-way ANOVA (*p* < 0.05).

In blood incubated at 37°C, the expression level of CD163 decreased continuously with increased incubation time and reached a significantly (*p* < 0.05) lower value after 4 h of incubation (MFI 5,736 ± 181) in comparison to CD163 levels after 1 h (MFI 7,132 ± 629) and 2 h (MFI 8,266 ± 563) of incubation at the same temperature (Figures 6A,B). In contrast, monocytes from blood incubated at 37°C increased their MHC class II molecules expression levels continuously with increased incubation time and showed a significantly (*p* < 0.05) higher expression level after 4 h of incubation (MFI 19,164 ± 1,512) in comparison to 1 h (MFI 13,093 ± 669) and 2 h (MFI 16,073 ± 553) incubation time (Figure 6). For blood incubated at 41°C, CD163 expression level remained constant during all incubation times, resulting in significantly (*p* < 0.05) higher CD163 expression levels after 4 h incubation at 41°C (MFI 7,878 ± 417) compared to 37°C (MFI 5,736 ± 181) (Figure 6B). After 4 h incubation of blood at 41°C, monocytes decreased their expression level of MHC class II molecules to a significantly lower level (MFI 7,218 ± 1,520) in comparison to MHC class II expression after 1 h (MFI 14,454 ± 264) and 2 h (MFI 16,803 ± 450) incubation at the same temperature or after 4 h incubation at 37°C (MFI 19,164 ± 1,512) (Figure 6B).

To evaluate the impact of heat stress on the response of monocytes to stimulation with lipopolysaccharide (LPS), the expression levels of the monocytic markers CD163 and MHC class II molecules were compared between blood samples stimulated at 37 and 41°C for 4 h. For both heat treatments (37 and 41°C), LPS stimulation for 4 h resulted in decreased expression levels of CD163 with increased expression levels of MHC class II molecules (Figure 6C). While the expression level of CD163 on LPS-stimulated monocytes was lower in blood incubated at 41 than 37°C, MHC II expression was higher after incubation at 41°C compared to 37°C (Figure 6C).

### Impact of Heat Stress on the Expression Levels of Cell Adhesion Molecules on Neutrophils and Monocytes

**Figure 7:**
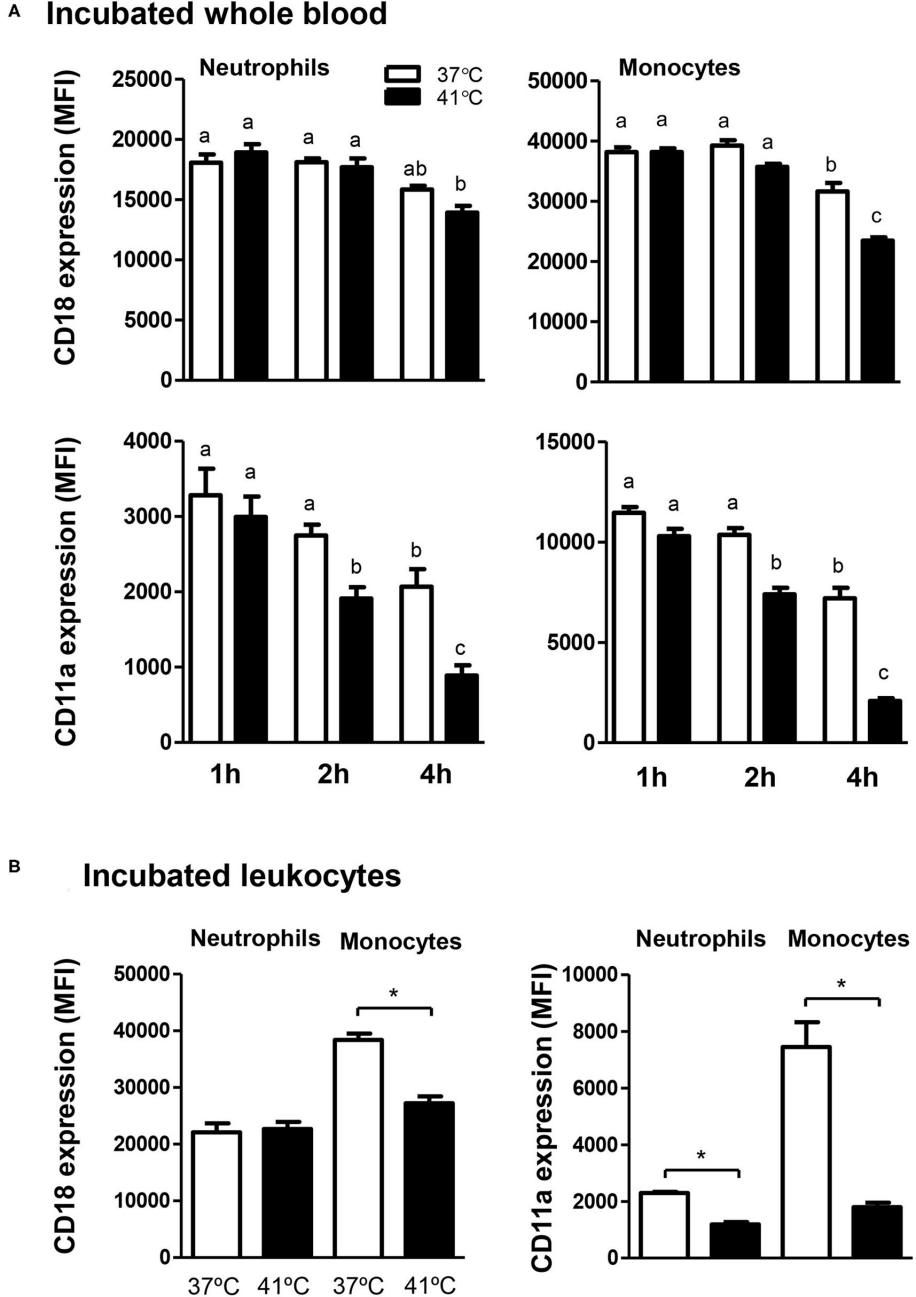
The effect of heat stress on adhesion molecules expression on camel neutrophils and monocytes. Leukocytes separated from heat-stressed whole blood **(A)** or heat-stressed leukocytes **(B)** were labeled with monoclonal antibodies to CD18 and CD11a and labeled cells were analyzed by flow cytometry. After setting gates on neutrophils and monocytes, the expression levels of CD18 and CD11a were calculated as MFI values and presented (mean ± SEM) for blood incubated at 37 and 41°C for 1, 2, and 4 h. Different lower case letters indicate a significant difference between the groups, as analyzed using one-way ANOVA (*p* < 0.05). For separated leukocytes, the student *t*-test was used for comparison between the means of cells incubated at 37 and 41°C (* indicates *p* < 0.05).

The expression level of the cell adhesion molecule CD18 on blood neutrophils and monocytes did not change significantly (*p* > 0.05) after 1 or 2 h incubation of whole blood at 37 or 41°C (Figure 7A). Incubation of whole blood for 4 h resulted in a significant (*p* < 0.05) decrease in the expression level of CD18 on neutrophils and monocytes when the blood was incubated at 41°C and only on monocytes when the blood was incubated at 37°C. In addition, after 4 h incubation at 41°C, monocytes showed a lesser abundance of CD18 (*p* < 0.05) compared to 37°C (Figure 7A). The abundance of CD11a on neutrophils and monocytes decreased gradually with increased incubation time (Figure 7A). For blood incubated at 37°C, the decrease in CD11a level was only significant (*p* < 0.05) after 4 h compared to 1 and 2 h incubation (Figure 7A). Incubation of whole blood at 41°C, however, induced a stronger decrease in CD11a expression levels on neutrophils and monocytes with significantly lower values after 2 h compared to 1 h of incubation. Incubation at 41°C for 4 h induced a further decrease in CD11a expression on neutrophils and monocytes with significantly (*p* < 0.05) lower levels compared to cells incubated for 2 h at the same temperature or for 4 h at 37°C (Figure 7A). When separated leukocytes instead of whole blood were used for heat treatment, similar changes in the expression pattern of the cell adhesion molecules CD11a and CD18 were observed (Figure 7B). After incubation of leukocytes at 41°C for 4 h, CD11a expression (for neutrophils and monocytes) and CD18 expression (only for monocytes) were significantly lower in comparison to cells incubated at 37°C for the same time.

## Discussion

Dromedary camels are known for their well-adaptation to the extreme hot desert environment, with the capability to elevate their body temperature to 41–42°C during severe hot (2, 3, 5, 6). In addition, camels, in comparison to other species from the same geographical area, show higher resistance to several infectious diseases (2, 18, 39–42). This suggests the existence of specific adaptation mechanisms of the camel immune system that allow their survival and higher resistance to diseases. This study was undertaken to assess whether *in vitro* treatment of camel blood at 41°C, simulating conditions of heat stress, differently alters cell vitality, phenotype, and function of leukocytes, compared to incubation at 37°C (normothermia).

As the heat-stress-induced changes were mainly observed after 4 h incubation of whole blood at 41°C and to exclude a possible impact of the separation procedure used for leukocyte separation from heat-stressed blood on the obtained results, leukocytes were firstly separated from blood and were then subjected to heat stress for 4 h. The comparable effects of heat treatment on separated leukocytes and whole blood samples, regarding leukocyte vitality, phagocytosis, ROS production, and cell adhesion molecules expression indicate no significant impact of the cell separation method on the results of the current study.

Although heat treatment of camel blood at 41°C did not induce cell necrosis in leukocytes, the fraction of apoptotic cells within the granulocytes, lymphocytes, and monocytes increased after 4 h incubation at 41°C. The higher increase in apoptotic granulocytes and monocytes compared to lymphocytes, however, suggests higher resistance of camel lymphocytes than granulocytes and monocytes to heat stress. This seems in line with the reported higher resistance of camel lymphocytes to heat stress compared to human lymphocytes (8). The comparison of the results of the current study with studies conducted previously on bovine leukocytes reveals similar pro-apoptotic effect of heat stress on camel and bovine monocytes (43). However, the pro-apoptotic effect of heat stress on camel neutrophils seems different from the reported thermotolerance of bovine neutrophils, which did not increase their apoptosis rate when exposed to *in vitro* heat stress (44).

Studies in different species have shown that heat stress can profoundly alter both the phenotype and function of several immune cells (45–47). Phagocytosis and production of reactive oxygen species (ROS) are key effector functions of phagocytes like neutrophils and monocytes, contributing mainly to bacterial clearance during infection (19). In the current study, incubation of camel blood at 41°C (compared to 37°C) for 4 h increased the phagocytosis activity and phagocytosis capacity of camel neutrophils and monocytes toward *S. aureus* and enhanced their ROS response to *in vitro* stimulation with *S. aureus*. This effect seems in contrast to the reported inhibitory effect of heat stress on phagocytosis and ROS production activities of bovine neutrophils (44). To investigate whether the improved antimicrobial functions of camel phagocytes during incubation under heat stress condition represent a thermotolerance and adaptation mechanism of the camel immune system, comparative immunologic studies involving cells from cattle and camels are required for uncovering the species-specific heat-stress adaptation mechanisms. The enhanced phagocytosis activity of neutrophils in heat-treated blood, compared to separated leukocytes, may be related to the indirect effect of heat shock on other cellular (platelets) or soluble (cytokines) factors in blood. This hypothesis, however, needs to be tested in further studies.

The cell surface molecules CD163 and MHC class II molecules are characteristic for the phenotype of monocytes and monocyte-derived macrophages (26, 27). In blood incubated under normal temperature, the continuous decrease in CD163 expression together with the increase in MHC class II molecules on monocytes indicates the early differentiation of camel monocytes toward macrophages with a classical M1 phenotype. In contrast, monocytes decreased their expression level of MHC class II molecules but did not change their CD163 expression level during the incubation of blood at 41°C, resulting in a CD163^high^ MHC-II^low^ phenotype resembling M2 macrophages phenotype (26, 48–50). Similar polarizing effect of heat stress has been recently reported for bovine monocytes (43). The exposure of bovine monocytes to high temperatures (41°C) guided their *in vitro* polarization from a classical activated M1 to a non-classically activated M2 phenotype (43). This is also confirmed by the infiltration of CD163^high^ myeloid cell population with monocytic origin into the intestinal mucosa and submucosa of cows exposed to heat stress (51). As heat-stressed neutrophils induced the development of an anti-inflammatory phenotype of human monocyte-derived macrophages (52), further investigations using separated cells are required to see whether heat stress directly modulated the phenotype of camel monocytes or indirectly via heat-stressed neutrophils in blood. The modulated expression levels of CD163 and MHC class II molecules on camel monocytes although indicates a polarizing effect of heat stress toward M2 macrophages, the detailed characterization of camel macrophage subtypes requires further functional studies.

To evaluate the impact of heat stress on the response of monocytes to LPS stimulation, the expression level of the monocytic markers CD163 and MHC class II molecules were compared between blood stimulated at 37 and 41°C. Overall, the increased expression of MHC class II molecules with the decrease in CD163 expression indicates the inflammatory phenotype of LPS-stimulated monocytes. When LPS-stimulated blood was incubated at 41°C, however, monocytes showed higher expression of CD163 and lower expression of MHC class II molecules, indicating an inhibitory effect of heat stress on the LPS-induced phenotypic response in camel monocytes.

The cell adhesion molecules CD18 and CD11a play essential roles during the different stages of adhesion and migration of blood leukocytes (19). In the present study, *in vitro* incubation of camel blood at 41°C induced a reduced expression of both CD18 and CD11a on neutrophils and monocytes. Whether this effect is associated with a reduced migration of neutrophils and monocytes in camels, still to be investigated using cell migration studies.

## Conclusions

The present study identified some heat-stress-induced phenotypic and functional alterations in camel blood leukocytes with similarities and differences from other species. Although heat treatment at 41°C for 4 h increased the fraction of apoptotic cells within all camel leukocyte subpopulations, the higher increase in apoptotic granulocytes and monocytes compared to lymphocytes suggests higher resistance of camel lymphocytes to heat stress compared to granulocytes and monocytes. Heat stress enhanced the phagocytosis and ROS production activities of camel neutrophils and monocytes toward *S. aureus*, which is in contrast to the reported inhibitory effect of heat stress on antimicrobial functions of bovine neutrophils. The phenotypic analysis of monocytes in camel blood incubated at 41°C indicates the development of CD163^high^ MHC-II^low^ monocytes, indicating a polarization toward anti-inflammatory M2 phenotype. In addition, heat stress treatment showed an inhibitory effect on the LPS-induced changes in camel monocytes phenotype. Furthermore, *in vitro* incubation of camel blood at 41°C reduced the expression of the cell adhesion molecules CD18 and CD11a on neutrophils and monocytes. Finally, the camel immune system and its adaptation to heat stress represents, in relationship to the ongoing global warming and increased droughts incidence, a valuable model for exploring the biological adaptations of the mammalian immune system to environmental changes. Further studies are needed to compare the species-specific thermotolerance of the immune system in camel and other animal species, including cattle, reared under the same environmental conditions.

## Data Availability Statement

The raw data supporting the conclusions of this article will be made available by the authors, without undue reservation.

## Ethics Statement

The animal study was reviewed and approved by The Ethics Committee at King Faisal University, Saudi Arabia (Permission number: KFU- KFU-REC/2020-09-25).

## Author Contributions

JH designed the work, collected the samples, performed the experiments, and prepared the manuscript.

## Conflict of Interest

The author declares that the research was conducted in the absence of any commercial or financial relationships that could be construed as a potential conflict of interest.
